# *In vitro* modeling of feline gut fermentation: a comprehensive analysis of fecal microbiota and metabolic activity

**DOI:** 10.3389/fmicb.2025.1515865

**Published:** 2025-01-29

**Authors:** Qianle Ren, Yuling Li, Mingmei Duan, Jinjun Li, Fangshu Shi, Yun Zhou, Wanjing Hu, Junfu Mao, Xiaoqiong Li

**Affiliations:** ^1^State Key Laboratory for Managing Biotic and Chemical Threats to the Quality and Safety of Agro-products & Food Sciences Institute, Zhejiang Academy of Agricultural Sciences, Hangzhou, China; ^2^College of Veterinary Medicine, Jilin University, Changchun, China; ^3^Guangzhou MYBAO Biotechnology Co., Ltd, Guangzhou, China; ^4^New Ruipeng Pet Group Inc., Beijing, China

**Keywords:** cat, gut microbiota, *in vitro* fermentation, targeted metabolites, SCFAs

## Abstract

The gut microbiota (GM) is a large and diverse microbial community that plays essential roles in host health. The *in vitro* fermentation model of the fecal GM serves as a valuable complement to food and health research in both humans and animals. Despite advancements in standardized protocols for culturing human GM, research concerning animals—particularly companion animals—remains limited. This study aims to identify the optimal *in vitro* fermentation method for cat gut microbiota by comprehensively analyzing fecal microbiota and fermentation characteristics. We evaluated seven culture media previously used to simulate the gut microenvironment in humans, dogs, and cats: anaerobic medium base (AMB), Minimum medium (MM), Pet medium (PM), VI medium (VI), VL medium (VL), Yeast culture medium (JM), and yeast casitone fatty acid agar medium (YCFA). Fresh fecal samples were fermented in these media for 48 h, followed by 16S rRNA sequencing to assess bacterial community composition and targeted metabolite monitoring during fermentation. The results revealed that the substrate composition in the medium differentially impacts bacterial community structure and fermentation characteristics. High levels of carbon and nitrogen sources can substantially increase gas production, particularly CO_2_, while also significantly enhancing the production of short-chain fatty acids (SCFAs). Additionally, substrates with a high carbon-to-nitrogen ratio promote the production of more SCFAs and biogenic amines, and enrich the *Bacteroidaceae* family, even when the total substrate amount is lower. Comprehensive analysis of gut microbiota and metabolites reveals that PM medium effectively simulates a nutrient-deficient microenvironment in the cat gut during *in vitro* fermentation. This simulation maintains bacterial community stability and results in lower metabolite levels. Therefore, using PM medium to culture cat gut microbiota for 48 h, without focusing on specific bacterial genera, represents the most suitable *in vitro* model. This finding contributes to understanding the optimal conditions for simulate cat gut microbiota and may provide a new approach for investigating the food pharmaceuticals on the cat gut microbiota and related health.

## 1 Introduction

Cats are among the most popular companion animals, and their health has become an increasing concern for pet owners (Wu et al., 2024). The GM, a complex microbial community involved in barrier protection, nutrition, metabolism, and immunity, is integral to host health (Li et al., 2024). It maintains gut and host homeostasis by defending against intestinal pathogens, providing nutrients, promoting nutrient digestion and absorption, enhancing barrier function, stimulating intestinal development, and regulating the immune system (Paone and Cani, 2020; Hill and Round, 2021; de Vos et al., 2022). Like those in the human microbiome, the key phyla in the microbiota of cats and dogs include *Firmicutes*, *Bacteroidetes*, *Proteobacteria*, *Actinobacteria*, and *Fusobacteria*. These microbes ferment dietary fiber and unabsorbed carbohydrates to produce lactic acid and short-chain fatty acids (SCFAs) while also generating gases such as carbon dioxide and hydrogen (Swanson et al., 2011; Vázquez-Baeza et al., 2016; Coelho et al., 2018). SCFAs, primarily acetate, propionate, and butyrate, serve as energy substrates for colonic epithelial cells, maintain epithelial barrier integrity, regulate energy metabolism, and exhibit anti-inflammatory effects, thereby contributing to gut and host health (Arpaia et al., 2013; Koh et al., 2016; Zhang et al., 2021). Conversely, bacterial fermentation of proteins through amino acid decarboxylation produces various biogenic amines (BAs), such as serotonin, putrescine, cadaverine, histamine, and tyramine, which pose significant toxicological risks (Lau et al., 2022).

Animal experiments are commonly used to study changes in and functions of the GM. For example, animal models have been established to investigate the effects of high-fructose corn syrup on skeletal health and the GM in male mice (Han et al., 2022), explore whether resveratrol alleviates type II diabetes by modulating the GM (Hou et al., 2023), and evaluate the antiaging effects of the dietary dye morin (Shenghua et al., 2020). However, conducting animal experiments is expensive and ethically controversial. Thus, *in vitro* fermentation has been utilized as an effective tool for assessing drug effects and conducting health-related research (Zhou et al., 2018; Yousi et al., 2019; Van den Abbeele et al., 2020).

Limitations in studying human and animal GM include difficulties in accessing the gut, complexity in microbial analysis, and ethical concerns. *In vitro* fermentation can address these challenges and provide an alternative research method (Wan et al., 2021). Establishing a stable *in vitro* cultivation method that maintains the bacterial community structure during drug experiments is crucial. Previous research has shown that MM medium, a peptone-containing oligotrophic fermentation medium, serves as a static batch fermentation model for human fecal samples and has been used to study the effects of single foods or nutrients on the composition and function of the GM (Pérez-Burillo et al., 2021). YCFA medium was used to culture chicken cecal contents, selectively enriching Desulfovibrionaceae (Crhanova et al., 2019). JM was used as an *in vitro* colonic model for dogs and cats to evaluate the effects of different yeast-derived formulations on the GM composition and metabolites (Van den Abbeele et al., 2020). Lei et al. (2012) used VL and VI media to cultivate human and chicken GM, respectively, analyzed differences in microbial composition and fermentation metabolites, and investigated the relationship between GM composition and function. AMB medium was identified as suitable for cultivating human GM *in vitro* (Wan et al., 2021). Like JM, PM has been used for *in vitro* fermentation experiments in dogs and cats to study the impact of different dietary fiber sources on microbial fermentation activity (Sunvold et al., 1995).

Studies indicate that approximately 50 to 90% of the GM can be cultured *in vitro* under suitable nutritional conditions. The addition of specific nutritional components or pharmaceutical ingredients can selectively culture certain bacterial taxa (Browne et al., 2016; Lau et al., 2016). *In vitro* gut models are categorized into simple static single-chamber models and more complex dynamic multichamber models. The thermostatic batch culture system, a widely used simple colon model, offers advantages such as ease of operation, no need for nutrient replenishment, and the ability to culture microbial communities in large batches (Deschamps et al., 2022). However, it is constrained by substrate availability, which limits the culture duration. In contrast, continuous fermentation dynamic models can consist of either single or multiple connected chambers. These models allow for the monitoring of parameters such as temperature, pH, and transit time in various gut regions, including the colon, while continuously replenishing substrates to sustain the GM over extended periods (from several weeks to months) (Fehlbaum et al., 2015). Despite these benefits, continuous fermentation models are more susceptible to contamination and are more challenging to use. Various media are used as *in vitro* alternatives for humans and animals, but comprehensive evaluations of the effectiveness of these media in simulating the gut environment are lacking. In this study, we inoculated fecal samples from healthy pet cats into the aforementioned seven culture media and analyzed the GM structure, SCFAs, and targeted metabolite levels after 48 h of fermentation. By comprehensively analyzing the GM and metabolites during cultivation, we determined the optimal *in vitro* fermentation medium for cat GM. Hence, an optimal fermentation medium should accurately simulate the growth environment of a cat’s GM while preserving its original community structure. The findings of this study could serve as a valuable reference for conducting *in vitro* cultivation experiments on cat fecal GM.

## 2 Material and methods

### 2.1 Reagents and materials

The SCFA standard solutions, including butyric acid, acetic acid, propionic acid, butanoic acid, isobutyric acid, valeric acid, and isovaleric acid, were obtained from Shanghai Aladdin Bio-Chem Technology Co., Ltd (Shanghai, China). Standards for eight BAs or their hydrochlorides—serotonin, phenylethylamine, spermine, agmatine, cadaverine, putrescine, octopamine, tyramine, and histamine—were purchased from Sangon Biotech (Shanghai, China). YCFA broth was acquired from Qingdao Haibo Biotechnology Co., Ltd (Shandong, China). Anaerobic medium base (AMB), minimum medium (MM), pet medium (PM), VI medium (VI), VL medium (VL), and yeast culture medium (JM) were prepared in house on the basis of prior research conducted in our laboratory. All other reagents used were of analytical grade.

### 2.2 *In vitro* culturing of the intestinal microbiota

Seven types of culture media—MM, VL, VI, PM, YCFA, JM, and AMB—were selected to simulate the intestinal environment. The compositions of these media are detailed in Supplementary Table 1. The constituents of the seven culture media were classified into eight distinct categories: sugars, nitrogen sources, vitamins, inorganic salts, minerals, and mucin. The visualization of bubble sizes corresponded to the concentration of each nutrient category (g/L), thereby enabling a detailed analysis of the effects and interactions of various nutrients on the bacterial community (Tramontano et al., 2018; Yousi et al., 2019). Data visualization was conducted utilizing ChiPlot. Media were prepared following published methods with minor modifications (Sunvold et al., 1995; Lei et al., 2012; Crhanova et al., 2019; Van den Abbeele et al., 2020; Wan et al., 2021). Each broth sample was added to a shaking tube.

Fecal samples were collected from nine healthy pet cats, with the uncultured original fecal group denoted the OR group. Information about the cats is provided in Supplementary Table 2, and this study was conducted with the consent of the cat owners. Approximately 2 g of each sample was placed in 20 mL of sterile physiological saline (0.9%, w/v), sealed with liquid paraffin, and transported on dry ice, with experiments conducted within 3 h. Subsequent experiments were performed in an anaerobic workstation (HYQX-III-Z, Shanghai YOKE Medical Instrument Co., Ltd., Shanghai, China) with an anaerobic gas mixture of 5% H_2_, 5% CO_2_, and 90% N_2_. After the samples were allowed to stand under anaerobic conditions for a few minutes, 1 mL of the supernatant was collected from beneath the liquid paraffin layer and added to a shaking tube containing 10 mL of broth. The culture media were connected to anaerobic gas through shaking tubes. Each broth was replicated three times, with unvaccinated broth serving as a control. All shaking tubes were cultured under anaerobic conditions at 37°C for 96 h with shaking at 100 rpm.

### 2.3 Sample collection

At 0, 24, and 48 h of fermentation, three sets of cultures were collected, rapidly frozen in liquid nitrogen, and stored at −80°C for subsequent analyses. For each time point, nine sample tubes were collected for gas production analysis, BA determination, pH measurement, ammonia analysis, SCFA analysis, and 16S rRNA sequencing.

### 2.4 Gas production analysis

After 48 h of fermentation, gas production and the levels of H_2_, CO_2_, CH_4_, and H_2_S were determined via a gas analyzer (HL-QT01, Hangzhou Hailu Medical Technology Co., Ltd.). The detection chamber was adjusted to a specific vacuum level via a vacuum generator, and the gases in the sample container were monitored by the 315 sensor in the detection chamber. The software (Multi-Gas Analyzer.exe) was used with a nonfecal medium as the zero gas to calculate the gas content and composition.

### 2.5 Determination of BAs

The assessment method for BAs in fermentation liquid samples was adapted from Sang et al. (2020) with modifications. Specifically, 100 μL of 2 mol/L sodium hydroxide solution, 300 μL of saturated sodium bicarbonate solution, and 2 mL of dansyl chloride derivatization reagent were sequentially added to 1 mL of pretreated samples. The mixture was incubated at 40°C in the dark for 45 min, after which 100 μL of concentrated ammonia was added to terminate the reaction. After standing for 30 min, the mixture was adjusted to volume with acetonitrile. The supernatant was filtered through a 0.22 μm organic phase membrane. For untreated fecal samples, PBS was used for dissolution. After centrifugation at 3,000 × *g* for 5 min at 4°C, the supernatant was processed as described for fermentation. BA quantification was performed via a Shimadzu high-performance liquid chromatography system with two elution solutions: A (ultrapure water, 0.1% acetic acid) and B (acetonitrile, 0.1% acetic acid). An automatic sampler passed through a ChromCore C18 column (4.6 × 250 mm, 5 μm) at a flow rate of 0.80 mL/min and a column temperature of 30°C, with ultraviolet detection at 254 nm; the injection volume was 10 μL. The gradient elution program was as follows: 0–2 min, 45% A; 2–20 min, 45–18% A; 20–27 min, 18–5% A; 27–30 min, 5% A; 30–32 min, 5–45% A; and 32–42 min, 45% A. Calibration curves were generated by analyzing a standard BA mixed solution for quantifying each BA.

### 2.6 Determination of pH value

At 0, 24, and 48 h of fermentation, 0.5 mL samples of fermentation broth were collected, and fermentation was stopped by immersing the samples in an ice–water bath for 10 min. The pH values of the fermentation broth were subsequently measured via a pH-5S meter (Shanghai Sansi Instrument Factory, Shanghai, China).

### 2.7 Ammonia and SCFA analysis

A fully automatic biochemical analyzer (HB-21, Suzhou Hailu Biotechnology Co., Ltd., China) was used to analyze fermentation liquid samples collected 48 h postfermentation with an ammonia detection reagent kit (enzyme method) from Zhejiang Ningbo Meikang Biotechnology Co., Ltd., China.

Five hundred microliters of fermentation broth was mixed with 100 microliters of butyric acid solution at a 1:5 ratio for acidification for 24 h, followed by storage at −80°C. Prior to SCFA determination, the samples were thawed and centrifuged at 14,000 rpm for 5 min, after which the supernatant was filtered through a 0.22 μm membrane. A gas chromatograph (GC-2010 Plus; equipped with a DB-FFAP column from Agilent Technologies, Inc., Santa Clara, CA, USA) was used for sample analysis.

### 2.8 DNA extraction, 16S rDNA amplicon library preparation and HiSeq sequencing

Bacterial DNA was extracted from the fecal samples via a fecal DNA kit (Omega Biotek, Norcross, GA, USA). The DNA concentration and purity were quantified with a NanoDrop 2000 UV-Visible spectrophotometer (Thermo Scientific, Wilmington, USA). DNA integrity was assessed by 1% agarose gel electrophoresis. The V3-V4 hypervariable region of the 16S rRNA gene was amplified via the gene-specific primers 338F (5′-ACTCCTACGGGAGGCAGAG-3′) and 806R (5′-GGACTACCVGGGTATCTAAT-3′). The PCR mixture included 10 μL of template DNA, 2 μL of ddH_2_O, 3 μL of primers, and 15 μL of Phusion High-Fidelity PCR Master Mix (New England Biolabs).

The PCR products were extracted from a 2% agarose gel and purified via the AxyPrep DNA Gel Extraction Kit (Axygen Biosciences, Union City, CA, USA). The purified DNA was quantified via a QuantiFluor-ST fluorometer (Promega, USA). Sequencing of the amplicons with equimolar and paired-end libraries was performed on the HiSeq 2500 platform (Illumina, CA, USA) following standard protocols provided by Shanghai Majorbio Pharmaceutical Technology Co., Ltd.

The raw paired-end sequencing data were quality controlled via fastp software^1^ (version 0.19.6). FLASH software was employed to assemble the cleaned sequences. The optimized sequences resulting from quality control and assembly were denoised via the DADA2 plugin in QIIME 2 with default parameters. We excluded sequences corresponding to chloroplast and mitochondrial origins, as well as those with a relative abundance of less than 0.5% across all sequences, from our analysis. Furthermore, we conducted rarefaction analysis on each sample, normalizing the sequence counts to the minimum observed, with all samples standardized to a uniform count of 16,666 sequences per sample. Taxonomic classification of amplicon sequence variants (ASVs) was performed via the naive Bayes classifier in QIIME 2. The Shannon diversity and Chao indices were computed via mothur v.1.30. Principal coordinate analysis (PCoA) based on the Bray-Curtis distance metric was employed to visualize the distance matrix of microbiota across different culture media at the ASV level.

### 2.9 Statistical analysis

Using IBM SPSS Statistics 27 for statistical analysis, the Shapiro–Wilk test and Levene’s test were employed to assess data normality and homogeneity of variances, respectively. For normally distributed data with equal variances (pH changes during the 0–48 h fermentation process in JM, YCFA, MM, and PM groups), one-way analysis of variance (ANOVA) followed by Bonferroni *post hoc* tests was conducted. For normally distributed data with unequal variances (ammonia, total gas production and H_2_ production after 48 h of fermentation), Welch’s test and Games-Howell *post hoc* tests were applied. For non-normally distributed data (BAs, CO_2_, H_2_S, and CH_4_ production, as well as gut microbiota α-diversity after 48 h of fermentation), the Kruskal-Wallis test with multiple pairwise comparisons was used. Bonferroni correction was applied to adjust significance thresholds for multiple comparisons. A *p*-value of < 0.05 was considered statistically significant.

## 3 Results

### 3.1 Comparison of media composition

**FIGURE 1:**
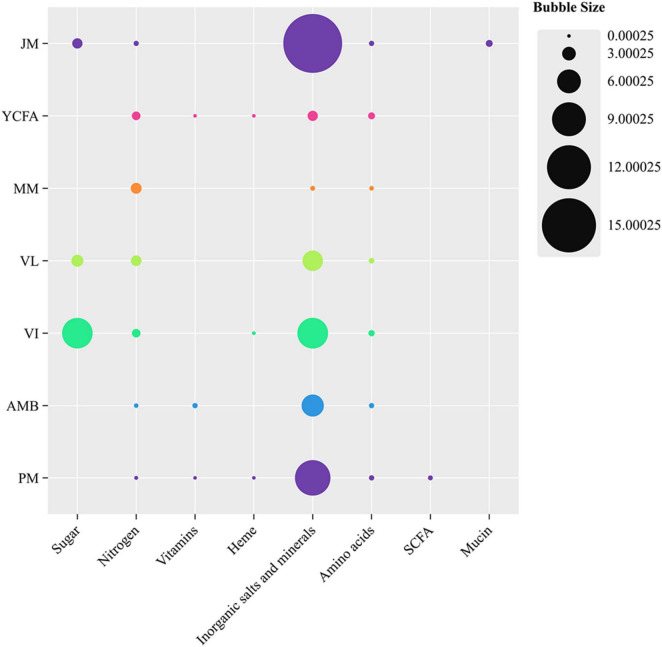
Comparison of different nutritional components in seven culture media: MM, AMB, VL, VI, YCFA, PM, and JM. The size of the bubbles is linearly related to the concentration of each nutritional category (g/L). ChiPlot was used for data visualization.

In this study, we employed seven culture media—MM, AMB, VL, VI, YCFA, PM, and JM—to cultivate the GM of cats. All media were composed of commercially available ingredients (Figure 1). MM lacks sugars and has the fewest nutritional components. The JM medium contains the highest contents of mucin, inorganic salts, and minerals. PM is the only medium supplemented with SCFAs as an energy source. VI and VL contain the highest amounts of sugars and relatively more nitrogen sources, inorganic salts, and minerals. The nutritional compositions of AMB and YCFA are similar; however, AMB has a lower nitrogen content and higher vitamin content than YCFA does.

### 3.2 Effects of microbial metabolic activity in terms of BAs, ammonia and gas production

**FIGURE 2:**
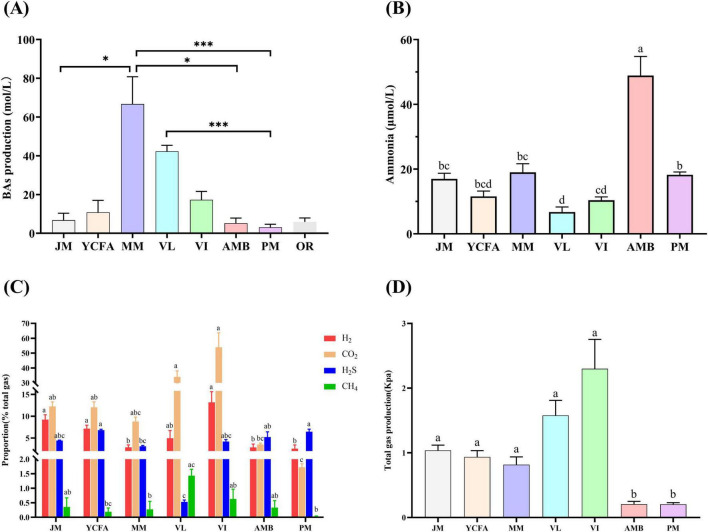
The production of BAs, ammonia and gas in different media after 48 h of fermentation. **(A)** BA production; compared with original feces OR, * indicates *p* < 0.05, *** indicates *p* < 0.001, no mark indicates that the difference was not significant. **(B)** Ammonia production; **(C)** proportions of H_2_, CO_2_, H_2_S and CH_4_ produced; **(D)** total gas production. Different letters indicate significant differences (*p* < 0.05), and the same letters indicate no significant difference (*p* > 0.05). The data are expressed as the means ± SEMs (*n* = 9).

Figure 2A shows the production of BAs from the seven culture media and the original fecal samples after 48 h of fermentation. BA production in the MM and VL groups was significantly greater than that in the control group OR (*p* < 0.001), whereas that in the JM, AMB, and PM groups was similar to that in group OR. We further evaluated the ammonia concentration in the fermentation broth of the seven culture media. As illustrated in Figure 2B, the ammonia concentration ranked from highest to lowest as follows: AMB > MM > PM > JM > YCFA > VI > VL, with significant intergroup differences (*p* < 0.05).

Additionally, Figure 2C shows the proportions of CO_2_, H_2_S, CH_4_, and H_2_ in each group. The VI group presented the highest proportion of CO_2_, whereas the PM group presented the lowest proportion, with significant differences between the groups (*p* < 0.05). The YCFA group had the highest proportion of H_2_S, while the VL group had the lowest, with significant differences (*p* < 0.05). The VI group presented the highest proportion of CH_4_, whereas the YCFA group presented the lowest proportion, with significant differences (*p* < 0.05). The VI group also had the highest proportion of H_2_, while the PM group had the lowest, with significant differences (*p* < 0.05). The total gas production after 48 h of fermentation ranked as follows: VI > VL > JM > YCFA > MM > AMB > PM, with significant differences among the groups (*p* < 0.05) (Figure 2D).

### 3.3 Overall microbial metabolic activity in terms of pH and SCFA production

**FIGURE 3:**
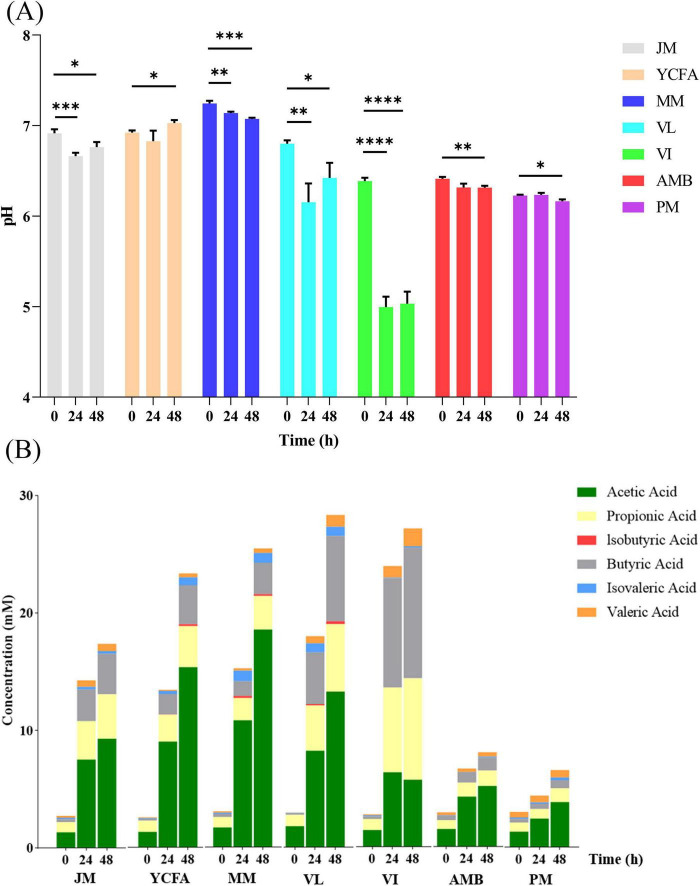
**(A)** Changes in pH in different media and times during *in vitro* culture; * indicates *p* < 0.05; ** indicates *p* < 0.01; *** indicates *p* < 0.001; **** indicates *p* < 0.0001. The data are expressed as the means ± SEMs (*n* = 9). **(B)** SCFA levels in different media and at different times during *in vitro* culture. The data are expressed as the means (*n* = 9).

Changes in pH can indicate the production of specific metabolites, primarily a decrease in pH due to acidic metabolites (e.g., SCFAs) and an increase in pH due to protein hydrolysis fermentation metabolites (e.g., BAs) (Van den Abbeele et al., 2020). During the simulation of the feline GM, with the exception of YCFAs, the pH values of the other media significantly decreased throughout the fermentation process compared with those before fermentation (0 h) (Figure 3A). The major decline occurred between 24 and 48 h, suggesting increased bacterial metabolic activity and the production of acidic metabolites.

SCFAs are crucial metabolic byproducts in the fermentation process and are primarily generated through the fermentation of indigestible carbohydrates (Ma et al., 2024). We detected the levels of SCFAs in the seven different culture media during the 48-h fermentation process (0, 24, and 48 h). As shown in Figure 3B, total SCFA production increased with prolonged fermentation time, reflecting changes in pH values. The VL and VI groups presented the highest total SCFA production at 48 h (28.3 ± 0.42 mmol), followed by the MM group, whereas the PM group presented the lowest SCFA production (6.57 ± 0.76 mmol). These findings indicate that the composition of the culture media significantly influences SCFA production. Nutrient-rich media with high carbon and nitrogen sources promote the rapid growth of SCFA-producing bacteria, resulting in increased SCFA levels. Conversely, nutrient-deficient media (PM and AMB) slow bacterial growth, leading to lower SCFA levels.

### 3.4 Effects of various cultivation media on the GM community

**FIGURE 4:**
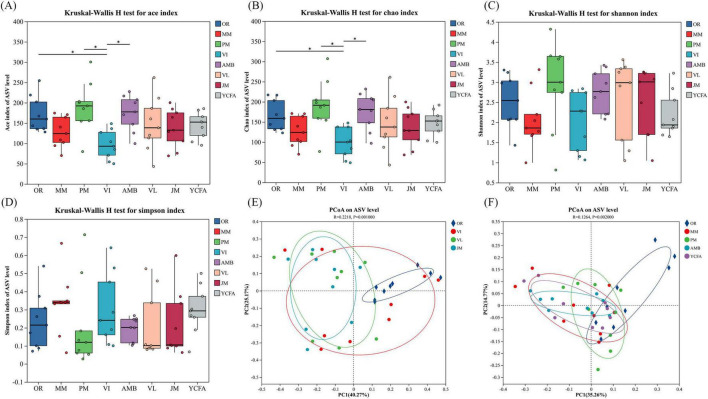
**(A–D)** Community diversity as measured by the Ace index **(A)**, Chao index **(B)**, Shannon index **(C)** and Simpson index **(D)** of the MM, AMB, JM, PM, VI, VL, YCFA, and OR groups after 48 h of fermentation. **(E,F)** Principal coordinate analysis (PCoA) between seven different media and the uncultivated group. * indicates *p* < 0.05. The data are expressed as the means ± SEMs (*n* = 9).

Cat fecal samples were inoculated with the seven tested media and fermented *in vitro* for 48 h. Sequencing was performed via the Illumina MiSeq platform, and the results were compared with those of OR to assess the effect of each medium on the gut flora. Alpha diversity, reflected by the Ace, Chao, Shannon, and Simpson indices, estimates microbial community diversity and abundance (Le Chatelier et al., 2013). Figures 4A–D show that the Chao and Ace indices of the VI group were significantly lower (*p* < 0.05) than those of the original cat feces M group. The Chao and Ace indices of the PM and AMB groups significantly increased, both of which were greater than those of the VI group (*p* < 0.05). The Simpson index was lowest in the PM group and highest in the MM group. The Shannon index was highest for the PM group and lowest for the MM group, but these differences were not significant.

To clearly present the PCoA results, we divided the analysis into two separate figures to compare the seven treatment groups with group OR. On the basis of nutritional components, different culture media were categorized into carbohydrate groups (VI, VL, JM) and nitrogen source groups (AMB, PM, YCFA, MM). PCoA describes the diversity of the GM among groups and compares it with that of OR. The PCoA results revealed that in the carbohydrate groups, the VI group exhibited a relatively large dispersion, indicating significant intragroup differences. The JM, VI, and VL groups were distinctly separated from the OR group, suggesting differences in community composition (Figure 4E). In the nitrogen source groups, there was no significant difference between the PM, YCFA, and MM groups and the OR group except for the AMB group, indicating a similarity in community composition. These data explain 35.26 and 14.77% of the variance in the PCoA model for PC1 and PC2, respectively (Figure 4F), suggesting the reliability of these profiles. Additionally, we employed Weighted UPGMA clustering analysis to observe the differences and similarities among various groups (Supplementary Figure 1). The results indicated that the PM group exhibited a high degree of similarity with the OR group, followed by the VI group. The remaining groups, however, showed significant differences from the OR group.

### 3.5 Composition of the GM

**FIGURE 5:**
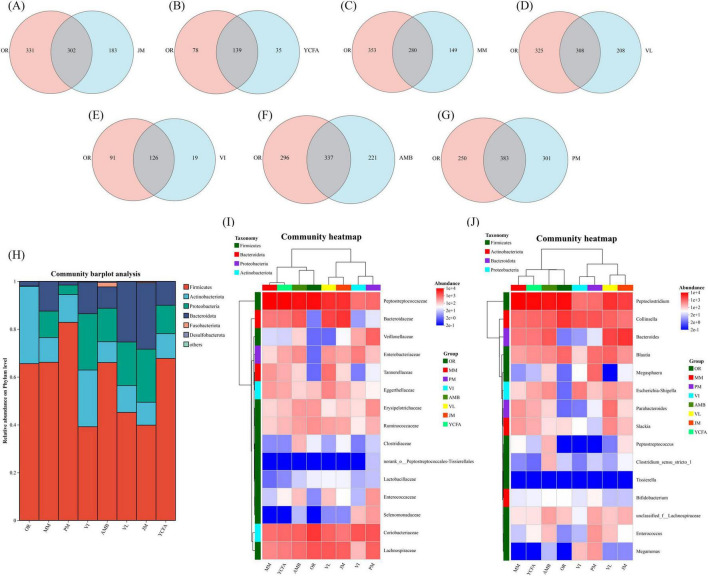
**(A–G)** Venn diagram showing the shared and unique numbers of ASVs between seven culture medium samples and the OR. **(H)** Stacked bar plot showing the average relative abundance at the phylum level. **(I)** Community heatmap showing the average relative abundance at the family level. **(J)** Community heatmap showing the average relative abundance at the genus level.

We used seven pairwise Venn diagrams to illustrate the retention of ASVs (Amplicon Sequence Variants) by the seven media compared to OR (Figures 5A–G). The results showed that PM medium shared 383 ASVs with OR, the highest among all groups. Additionally, PM uniquely retained 250 ASVs, while OR had 301 unique ASVs. The second-highest retention was observed in AMB medium, which shared 337 ASVs with OR. The other media exhibited relatively poorer retention performance. To further evaluate the cultivation effects of different media, we conducted an analysis of the microbial community composition and LEfSe differential analysis for the seven media groups and the control group. *Firmicutes*, *Actinobacteria*, *Proteobacteria*, and *Bacteroidota* were the dominant phyla in all groups, whereas *Fusobacteriota* accounted for a relatively small proportion in each group, indicating changes in the relative abundance of the top six phyla across groups (Figure 5H). The heatmap of community composition at the family level (Figure 5I) revealed that only the VI group presented a significant decrease in the content of *Lachnospiraceae* at 48 h compared with the uncultured group M. Groups MM, YCFA, and JM presented little change in the content of *Enterococcaceae* and *Enterobacteriaceae* at 48 h, whereas the content of *Coriobacteriaceae* and *Ruminococcaceae* decreased sharply. *Erysipelotrichaceae* significantly decreased in the MM and JM groups, whereas *Bacteroidaceae* significantly increased in the JM group. In the VL group, the contents of *Bacteroidaceae* and *Tannerellaceae* increased sharply, and *Tannerellaceae* remained relatively stable during incubation, except in the VL group. Microbial families with relatively few observations also showed significant changes, such as *Veillonellaceae, whose abundance* significantly increased in the PM group, and *Selenomonadaceae, whose abundance* significantly increased in the VI group. After 48 h of fermentation, the relative abundance of *Coriobacteriaceae* in six out of the seven culture media, with the exception of VI, was lower than that in the original unfermented fecal samples. This could be attributed to the anaerobic nature of bacteria belonging to the *Ruminococcus* genus, making cultivation challenging. Throughout the cultivation process, the relative abundances of *Enterococcaceae* and *Enterobacteriaceae* remained stable. The analysis of substrate composition revealed that the high carbon-to-nitrogen ratios in JM and VL significantly promoted the growth of *Bacteroidaceae*. Similarly, at the genus level (Figure 5J), comparable results were observed. The dominant and subdominant genera in OR, including *Peptoclostridium*, *Collinsella*, and *Blautia*, were retained in both PM and VL media, where they also remained dominant genera.

LEfSe (linear discriminant analysis effect size) analysis was used to compare the estimated bacterial phylotypes of the seven media at 48 h. Evolutionary maps were plotted via the default parameters (*p* < 0.05, LDA score > 2.0), which revealed differences in abundance between the groups (Supplementary Figure 2). In the OR group, *Clostridia*, *Erysipelotrichaceae*, *Erysipelotrichales*, *Prevotellaceae* and *Holdemanella* were the dominant classes of bacteria. In the PM group, *Lachnospirales*, *Lachnospiraceae*, *Veillonellaceae* and *Megasphaera* were dominant. In the VI group, *Negativicutes*, *Selenomonadaceae* and *Megamonas* were the most dominant. In the AMB group, *Peptostreptococcus*, *Fusobacteriia* and *Fusobacteriales* were dominant. In the VL group, *Tannerellaceae*, *Parabacteroides* and *Alistipes* were the most dominant. In the JM group, *Bacteroidales*, *Bacteroidia* and *Bacteroidaceae* were dominant. In the YCFA group, *Peptostreptococcaceae* and *Peptoclostridium* were the dominant classes of bacteria. The MM group is highly consistent with the YCFA group.

### 3.6 Functional predictions of the GM communities

**FIGURE 6:**
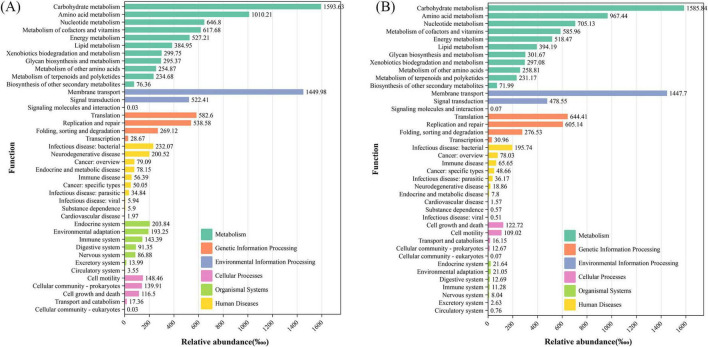
Tax4Fun function prediction of the PM-48 h group **(A)** and original feces **(B)** (the composition of the pathways of different components is displayed dynamically; the KEGG pathways of different levels are arranged on the vertical axis; and the length of the column indicates the corresponding functional abundance in the pathway).

For Tax4Fun functional prediction (Figure 6 and Supplementary Figures 3–8), the metabolic pathways of the eight groups were similar, encompassing carbohydrate metabolism, amino acid metabolism, and energy metabolism. Environmental information processing, genetic information processing, cellular processes, human diseases, and organismal systems are also consistent, with subtle variations in values.

### 3.7 Correlation analysis between metabolites and the GM

We investigated the correlations among SCFAs, BAs, ammonia, and the GM (Supplementary Figure 9). Species from the *Clostridiaceae* family (*Holdemanella*, *Peptococcus*) were significantly negatively correlated with the production of propionic acid and butyric acid. Conversely, bacteria from the *Bacteroidaceae* family (*Bacteroides*, *Slackia* and *Parabacteroides*) were associated with increased production of acetic acid, propionic acid and butyric acid. The correlation trends between total SCFAs and the GM, as well as between acetic acid-GM and butyric acid-GM, were generally consistent, as acetic and butyric acids are the primary SCFAs. Thus, these data explain the high accumulation rate of SCFAs after 48 h of fermentation in groups enriched with *Bacteroidaceae* and with reduced *Lachnospiraceae*, such as VL, VI, MM, YCFA, and JM. Compared with those in the PM group, the abnormal increase in *Bacteroidaceae* and decrease in *Lachnospiraceae* in these groups disrupted the initial balance of the GM, resulting in increased SCFA production. This significant imbalance impacts the stability and reliability of SCFA content determination in fermentation studies *in vitro*. Additionally, *Enterococcus* was significantly negatively correlated with CO_2_ production, *Megasphaera* was significantly positively correlated with H_2_S production, and *Enterococcus* and *Holdemanella* were significantly negatively correlated with H_2_ production. *Slackia* was negatively correlated with the production of ammonia and BAs, whereas *Escherichia-Shigella* had the opposite effect.

## 4 Discussion

While research on optimizing *in vitro* fermentation protocols for the human GM is extensive (Van den Abbeele et al., 2018), few studies have focused on the GM of pets, especially cats. The primary objective of this study was to assess the impact of different anaerobic batch culture media on the GM of felines and their associated metabolic products. The ultimate goal was to identify the most suitable *in vitro* fermentation method for simulating the cat gut microbiome.

Throughout the fermentation process, pH variation serves as an indicator of the overall metabolic activity of the GM, which is influenced by both the composition of the medium and the metabolic characteristics of the microbiota. In this study, among the seven tested media, the MM group presented the highest nitrogen content, whereas the VI group presented the highest carbohydrate content. The GM utilizes nitrogen sources during fermentation, leading to the release of ammonium ions and an increase in pH (Cummings and Macfarlane, 1991; Ma et al., 2017). Conversely, carbohydrate metabolism leads to the generation of organic acids (Laparra and Sanz, 2010), causing a decrease in pH. These factors explained the observed pH values after 48 h of fermentation, with the MM group having the highest pH and the VI group having the lowest pH. During the 48-h fermentation process, the pH exhibited a similar trend across all seven media, suggesting the preliminary feasibility of culturing cat fecal microbiota.

Ammonia in the colon originates primarily from the deamination process of dietary or endogenous proteins. Previous studies have demonstrated that dietary protein supplementation significantly increases the ammonia concentration in the intestines and feces (Geypens et al., 1997). Excessive accumulation of ammonia in the intestines can have adverse effects on overall health, including the disruption of intestinal mucosal repair and an increased risk of colon cancer. In a healthy colon, ammonia levels are typically low (Williams et al., 2001). Conversely, increasing carbohydrate levels, which serve as a source of energy for fermentation and bacterial growth, can reduce intestinal ammonia concentrations (Williams et al., 2001). Consistent with these findings, our study revealed that the VL and VI groups, characterized by high sugar contents, presented the lowest ammonia production. In contrast, the JM, YCFA, MM, and PM groups presented slightly greater ammonia production, whereas the AMB group presented significantly greater ammonia production than did the other groups. Given that cats are obligate carnivores with a diet predominantly composed of nitrogen-rich sources, the use of AMB *in vitro* to study feline dietary health may lead to biased assessments of ammonia production.

BAs are low-molecular-weight organic nitrogen bases that naturally occur as metabolic intermediates and byproducts in living organisms. They play crucial roles in various physiological functions, such as brain activity, gastric acid secretion, and immune responses. However, excessive absorption of BAs can pose health risks (Önal et al., 2013; Butor et al., 2023). BAs can accumulate in food at high concentrations due to the activity of microorganisms with decarboxylase enzymes, and recent outbreaks of foodborne diseases caused by BAs underscore the importance of their detection (Butor et al., 2023).

SCFAs produced by the GM play a vital role in maintaining host health by inhibiting the growth of harmful bacteria through pH reduction (Morrison and Preston, 2016). SCFAs have garnered substantial attention because of their pharmacological and physiological properties and potential impact on host health (Rauf et al., 2022). In *in vitro* fermentation studies investigating the protective effects of probiotics and functional foods, SCFA concentrations are commonly measured. This study revealed that different media compositions resulted in varying microbial community structures and, subsequently, different SCFA levels. When functional foods were studied via *in vitro* experiments, the use of specific media, such as VL, VI, or MM, resulted in higher concentrations of SCFAs than other media. This poses a challenge in attributing the increased SCFA levels solely to the functional food components, as the media itself promotes SCFA production. Therefore, for *in vitro* fermentation studies associated with food research, media with lower SCFA concentrations, such as PM and AMB, are more appropriate.

Research on the composition of human intestinal gas has identified CO_2_, H_2_S, CH_4_, and H_2_ as the predominant gases produced primarily through GM fermentation (Ou et al., 2015). Among these gases, CO_2_ is a major byproduct of carbohydrate fermentation. Consistent with previous studies (Zhang et al., 2023), the VI group, which had a high carbohydrate content, presented the highest proportion of CO_2_. This suggests that the variations in CO_2_ proportions among the groups are largely influenced by the carbohydrate content of the media. The phyla *Firmicutes* and *Bacteroidota* play significant roles in gas production in the intestine, with H_2_ production being particularly common due to specific hydrogen-producing GM (Ou et al., 2015; Hylemon et al., 2018; Mutuyemungu et al., 2023). After 48 h of fermentation, the VI group presented the highest proportion of H_2_ production among the seven groups, indicating a relatively high abundance of these species. Moreover, H_2_ is utilized by other GMs to produce H_2_S and CH_4_. In this study, the YCFA group had the highest proportion of H_2_S production, suggesting a greater abundance of hydrogen sulfide-fermenting bacteria in this group. Additionally, the VL group had the highest proportion of CH_4_, although it was the smallest proportion among the seven groups, indicating a relatively lower presence of methane-fermenting microbiota in the feline colon. Under normal circumstances, the production of intestinal gas in the host is minimal. However, an imbalance in the proliferation of gas-producing bacteria can lead to excessive gas production, which can cause abdominal distension and discomfort (Martin-Gallausiaux et al., 2021). Intestinal gas production is closely associated with the pathogenesis of several gastrointestinal diseases and can be considered a biomarker for such conditions (Wang et al., 2021). Compared with the other groups, the PM and AMB groups presented relatively lower gas production, suggesting a more stable fermentation metabolism of the feline intestinal microbiota under these conditions.

After 48 h of cultivation, the microbial communities in the seven groups exhibited distinct changes. Compared with those of the original fecal GM, the Chao and Ace indices of the VI group significantly decreased, with the VL, MM, JM, and YCFA groups also showing slight decreases. In contrast, the Chao and Ace indices of the AMB and PM groups remained high, indicating that these groups effectively maintained community diversity and richness after 48 h of fermentation. PCoA revealed that the microbial community distribution in the original cat feces was similar to that in the PM, YCFA, and MM groups, whereas it significantly differed from that in the VI, VL, and JM groups. We speculate that, owing to differences between *in vivo* and *in vitro* environments and the inability of some bacteria to be cultured outside the body, all seven groups experienced varying degrees of ASV loss after 48 h of cultivation compared with the OR group. Additionally, PM and AMB retained the highest number of ASVs from the original feces, indicating that the PM and AMB effectively preserved the microbial diversity in the original cat fecal microbiota. Fermentation with AMB medium for 48 h effectively maintained the stability of the human intestinal microbial community structure, as reported in previous studies (Wan et al., 2021), which can be attributed to the different compositions of the human and cat intestinal microbial communities (Van den Abbeele et al., 2020). LEfSe analysis revealed differentially abundant taxa among the groups, with the GM community being more diverse in the PM medium, indicating that PM medium is suitable for most anaerobic bacteria. LDA scores revealed that *Lachnospiraceae* and *Tannerellaceae* were the bacterial taxa with significant differences in abundance between the PM and VL groups. Previous studies have shown that *Lachnospiraceae* and *Tannerellaceae* are primarily responsible for SCFA production (Zhao et al., 2022; Ćesić et al., 2023), suggesting a correlation between PM and VL media and high SCFA levels. The community composition results at both the family and genus levels indicated that the AMB and PM medium effectively preserved the dominant and subdominant microbiota from OR, without enriching low-abundance taxa in OR. In addition to AMB and PM, the results for YCFA also showed relatively small deviations from the OR group, and this smaller difference may be related to the poorer nutritional content of these media, resulting in lower modification of the microbiota composition. The observed changes in the gut flora across the groups were due mainly to differences in the media composition, leading to variations in metabolite levels, which is consistent with the SCFA analysis results. Correlation analysis suggested that the high accumulation rate of SCFAs, such as VL, VI, MM, YCFA, and JM, after 24 h of fermentation in groups enriched with *Bacteroidaceae* bacteria and reduced the abundance of *Lachnospiraceae* resulted in a significant imbalance, affecting the stability and reliability of SCFA content determination in *in vitro* fermentation studies.

An ideal *in vitro* gut fermentation model should simulate the *in vivo* conditions of cats. First and foremost, it must maintain a microbial community that is similar in both composition and structure to the selected gut compartment. Specifically, the *in vitro* microbial composition should mirror the inoculum or the simulated intestinal region, effectively preserving both dominant and subdominant genera. Given the vast number of species in feline fecal microbiota that are uncultured *in vitro* and the resolution limitations of 16S rRNA gene sequencing, comparisons are typically conducted at the family and genus levels (Zihler Berner et al., 2013). In our study, at the phylum level, the most abundant *Firmicutes* and the subdominant *Actinobacteriota* in the original feces were well-maintained in the PM medium as well as in other media. At the family level, the dominant *Peptostreptococcaceae*, *Coriobacteriaceae*, and *Lachnospiraceae* in the original feces also showed dominant abundance in the PM medium. Similarly, at the genus level, the dominant genera *Peptoclostridium*, *Collinsella*, and *Blautia* in the original feces and PM were preserved (Figures 5H–J). Although the microbiota structure within the AMB medium exhibited the closest clustering to the OR, the PM medium demonstrated a superior retention of ASVs present in the OR group, with a count of 383 compared to 337 for the AMB medium. This result underscores the PM medium’s efficacy in preserving a greater proportion of the microbial diversity inherent in the original cat fecal microbiota (Figures 5A–G). Additionally, the PM group registered the highest α-diversity among the seven experimental groups, as depicted in Figures 4A–C, and showed a closer resemblance to the OR in β-diversity analysis (Figure 4F). Collectively, these observations indicate that the PM medium outperformed the other six culture media in terms of maintaining the fidelity and diversity of the cat fecal microbiota.

Simultaneously, it is imperative to ensure that the metabolic activity of the microbiota in the *in vitro* model closely resembles that observed *in vivo*, particularly regarding the production of SCFAs, biogenic amine, ammonia, and gases during fermentation. The *in vitro* fermentation process should replicate the production of major SCFAs—namely acetic acid, propionic acid, and butyric acid—in proportions akin to those present in the inoculum (Isenring et al., 2023). Our study revealed that, after 48 h of fermentation, among the seven media tested, only the PM and AMB media generated a modest amount of SCFAs, with acetic acid being the predominant component, followed by propionic and butyric acids, mirroring the SCFA profile in the original feces at the commencement of fermentation (Figure 3B). Both the AMB and PM groups produced comparatively low levels of biogenic amine and gases. However, unlike the PM group, the AMB group yielded the highest amount of ammonia, which is detrimental when simulating colon fermentation conditions. Functional metagenomic prediction is instrumental in assessing the alignment between the metabolic and functional KEGG pathways of microbial communities within the *in vitro* fermentation model and their counterparts in the original microbial communities (Poeker et al., 2019). In our study, the metabolic pathways of the microbial communities across the seven media groups were found to be similar to those of the original fecal group, with only subtle numerical variations observed. Therefore, after a meticulous evaluation of these parameters, we propose that the PM medium is the most appropriate choice for *in vitro* fermentation models.

Furthermore, the chosen *in vitro* model must exhibit reproducibility. Acknowledging the substantial inter-individual variability inherent in gut microbiota, research on *in vitro* model selection should incorporate adequate replication and enhance the interpretability of data by leveraging microbiota from diverse donors. This approach addresses the individual variations within groups and broadens the generalizability of findings (Isenring et al., 2022). In line with this principle, we selected fecal microbiota from nine cats, representing a spectrum of genders, ages, and breeds, to serve as our inocula. This method circumvented the limitations associated with using samples from a single cat or pooling fecal samples for iterative cultivation experiments. Consequently, this strategy had been pivotal in ensuring the reproducibility and reliability of our study’s results.

*In vitro* gut fermentation, uncoupled from the host, offers an optimal system for examining microbial responses to nutritional interventions, as it allows for the measurement of microbial dynamics without the confounding influence of the host (Liu et al., 2020). Both simple static batch fermentation models and complex dynamic models have their respective merits and drawbacks (Deschamps et al., 2022). Batch models, when compared with continuous fermentation models, exhibit certain limitations, including an inability to sustain stable microbial communities derived from the inoculum over prolonged periods, a lack of real-time pH control, and an inefficient removal of metabolic waste (Duysburgh et al., 2020). Consequently, batch and continuous fermentation models demand distinct media requirements. Static batch fermentation is designed to provide short-term energy to the microbial community, without the need for nutritive growth medium renewal until the conclusion of the experiment (Gonza et al., 2024). These methods are therefore constrained by substrate availability over a limited timeframe (24 to 72 h), with parameters such as pH or redox potential remaining unregulated. Therefore, the transition from batch to continuous fermentation models necessitates several considerations. First, a nutritive medium that emulates ileal effluents, comprising a variety of complex carbon and nitrogen sources, electrolytes, BAs, and vitamins, must be continuously supplemented to the bioreactor and refreshed promptly to maintain the balance of microbiota and metabolic products, as well as to accommodate the growth rates of specific microbiota and the production rates of key metabolites (Xie, 2022). Second, the substrate concentration in the medium must be adjusted to fit the continuous supplementation and removal processes inherent to continuous fermentation models (Qin and Zhai, 2024). Thus, when applying our optimally selected PM medium from the batch fermentation model to continuous fermentation, it requires ongoing optimization based on actual microbiota growth conditions. Additionally, beyond controlling temperature and pH, simulating *in vivo* conditions requires careful consideration of retention time, which should be inferred from the digestion rate of food outside the host (Poeker et al., 2019; Qin and Zhai, 2024). Finally, during the post-inoculation stabilization period, fermentation parameters must be adjusted based on observations of fermentation broth color, pH, metabolic products, and 16S rRNA sequencing results, ensuring the preservation of the microbial community from the inoculum (Poeker et al., 2019). It is imperative to emphasize that *in vitro* models for human and animal intestinal microbiota research are merely representations of reality. Inaccurate handling, data analysis, and unsupported correlations with host health and disease treatment can compromise research validity (Isenring et al., 2023). Therefore, the limitations of both batch and continuous *in vitro* models should be meticulously considered.

## 5 Conclusion

After a 48-h fermentation period in which cat fecal samples were used as inocula, the Pet medium presented the lowest production of total SCFAs, a more stable bacterial community structure, and consistent physicochemical properties. Consequently, the PM effectively preserved the balance of the GM and serves as a suitable *in vitro* model for studying the batch fermentation of potential functional food components via the cat intestinal flora. This approach provides an expedited avenue for investigating the nutritional and health-related value of pet diets.

## Data Availability

The datasets presented in this study can be found in online repositories. The names of the repository/repositories and accession number(s) can be found in this article/Supplementary material.

## Footnotes

1
https://github.com/opengene/fastp
