# Supplementary material for: In vitro modeling of feline gut fermentation: a comprehensive analysis of fecal microbiota and metabolic activity
Source: Front Microbiol. 2025 Jan 29;16:1515865. doi: 10.3389/fmicb.2025.1515865 (PMC11813885; doi:10.3389/fmicb.2025.1515865)
Supplement: Supplementary file 1 [file Data_Sheet_1.docx]

***Supplementary material***

**Table S1** The composition of five culture medium (g/L).

**Table S2** Information about the cats in this study.

**Fig S1** Using hierarchical clustering tree to display species composition similarities and differences between samples

**Fig S2** The microbial communities were characterized through LEfSe analysis and LDA.

**Fig S3-S8** Tax4Fun function prediction of AMB-48h, JM-48h, MM-48h, VI-48h, VL-48h, YCFA-48h and M-48h Group.

**Fig S9** The microbial communities were characterized through LEfSe analysis and LDA.

**Table S1.** The composition of seven culture medium (g/L).

| **MM (pH 7.0±0.1)** | | **VL (pH6.4±0.1)** | | **VI (pH6.4±0.2)** | | **YCFA (pH6.9±0.1)** | | **PM (pH6.4±0.2)** | | **AMB (pH 7.5±0.1）** | | **JM (pH 6.9±0.1）** | |
| --- | --- | --- | --- | --- | --- | --- | --- | --- | --- | --- | --- | --- | --- |
| Peptone | 15 | Beef extract | 2.4 | Maltose | 8 | Tryptone | 10 | NaCl | 5.4 | K_2_HPO_4_ | 0.3 | K_2_HPO_4_ | 3.5 |
| Cysteine | 0.312 | Yeast extract | 5 | Tryptone | 3 | Yeast extract | 2.5 | KH_2_PO_4_ | 2.7 | KH_2_PO_4_ | 0.2 | KH_2_PO_4_ | 10.9 |
| Na_2_S | 0.312 | Glucose | 2.5 | Peptone | 3 | L-cysteine | 1 | CaCl_2_·6H_2_O | 0.16 | NaCl | 0.45 | NaHCO_3_ | 2 |
| NaOH | 0.08 | Tryptone | 10 | Yeast extract | 4.5 | Heme | 0.01 | MgCl_2_·6H_2_O | 0.12 | (NH_4_)_2_SO_4_ | 0.45 | Yeast extract | 2 |
| Resazurin | 0.00125 | L-cysteine hydrochloride | 0.6 | Bile salt | 0.4 | NaCl | 0.9 | MnCl_2_·4H_2_O | 0.06 | CaCl_2_ | 0.05 | Peptone | 2 |
|  |  | NaCl | 5 | L-cysteine hydrochloride | 0.8 | CaCl_2_·6H_2_O | 0.09 | CoCl_2_·6H_2_O | 0.06 | MgSO_4_ | 0.1 | Starch | 2 |
|  |  |  |  | Heme | 0.05 | KH_2_PO_4_ | 0.45 | (NH_4_)_2_SO_4_ | 5.4 | L-cysteine | 0.5 | Mucin | 1 |
|  |  |  |  | NaCl | 4.5 | K_2_HPO_4_ | 0.45 | K_2_HPO_4_ | 0.89 | L-ascorbic acid | 0.5 | Cysteine | 0.5 |
|  |  |  |  | KCl | 2.5 | MgSO_4_·7H_2_O | 0.09 | Thiamine | 0.1 | Na_2_CO_3_ | 4 |  |  |
|  |  |  |  | MgCl_2_·6H_2_O | 0.45 | Resazurin | 0.001 | D-pantothenic | 0.1 | Beef extract | 1 |  |  |
|  |  |  |  | CaCl_2_·6H_2_O | 0.2 | Vitamin I | 0.0005 | Niacin | 0.1 | Peptone | 1 |  |  |
|  |  |  |  | KH_2_PO_4_ | 0.4 | Biotin | 0.002 | VB6 | 0.1 | Nutrient agar | 1 |  |  |
|  |  |  |  | Uric acid | 0.7 | Cobalamin | 0.002 | Folic | 0.01 |  |  |  |  |
|  |  |  |  | Resazurin | 0.0005 | P-aminobenzoic acid | 0.006 | NH_4_HCO_3_ | 0.1 |  |  |  |  |
|  |  |  |  | MgSO_4_·7H_2_O | 6 | Folic acid | 0.01 | Riboflavin | 0.01 |  |  |  |  |
|  |  |  |  | MnCl_2_·4H_2_O | 0.64 | Pyridoxamine | 0.03 | Heme | 0.01 |  |  |  |  |
|  |  |  |  | FeSO_4_·7H_2_O | 0.2 |  |  | Resazurin | 0.001 |  |  |  |  |
|  |  |  |  | CoSO_4_·7H_2_O | 0.36 |  |  | Yeast extract | 0.5 |  |  |  |  |
|  |  |  |  | CaCl_2_·2H_2_O | 0.2 |  |  | Tryptone | 0.5 |  |  |  |  |
|  |  |  |  | ZnSO_4_·7H_2_O | 0.36 |  |  | Na_2_CO_3_ | 4 |  |  |  |  |
|  |  |  |  | CuSO_4_·5H_2_O | 0.02 |  |  | L-cysteine hydrochloride | 0.5 |  |  |  |  |
|  |  |  |  | NiCl_2_·6H_2_O | 0.184 |  |  |  |  |  |  |  |  |

**Table S2** Information about the cats in this study.

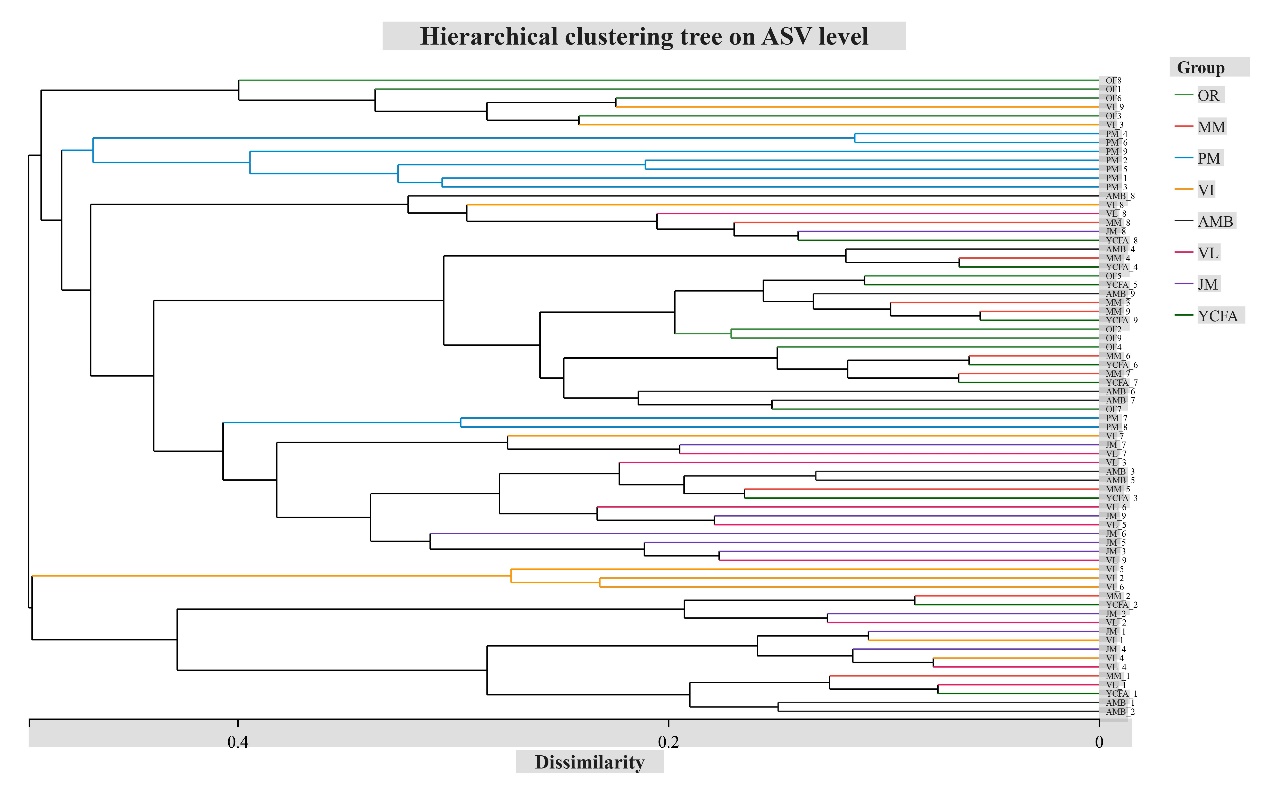


Fig S1 (A) Weighted UPGMA clustering analysis of the gut microbiota among groups.

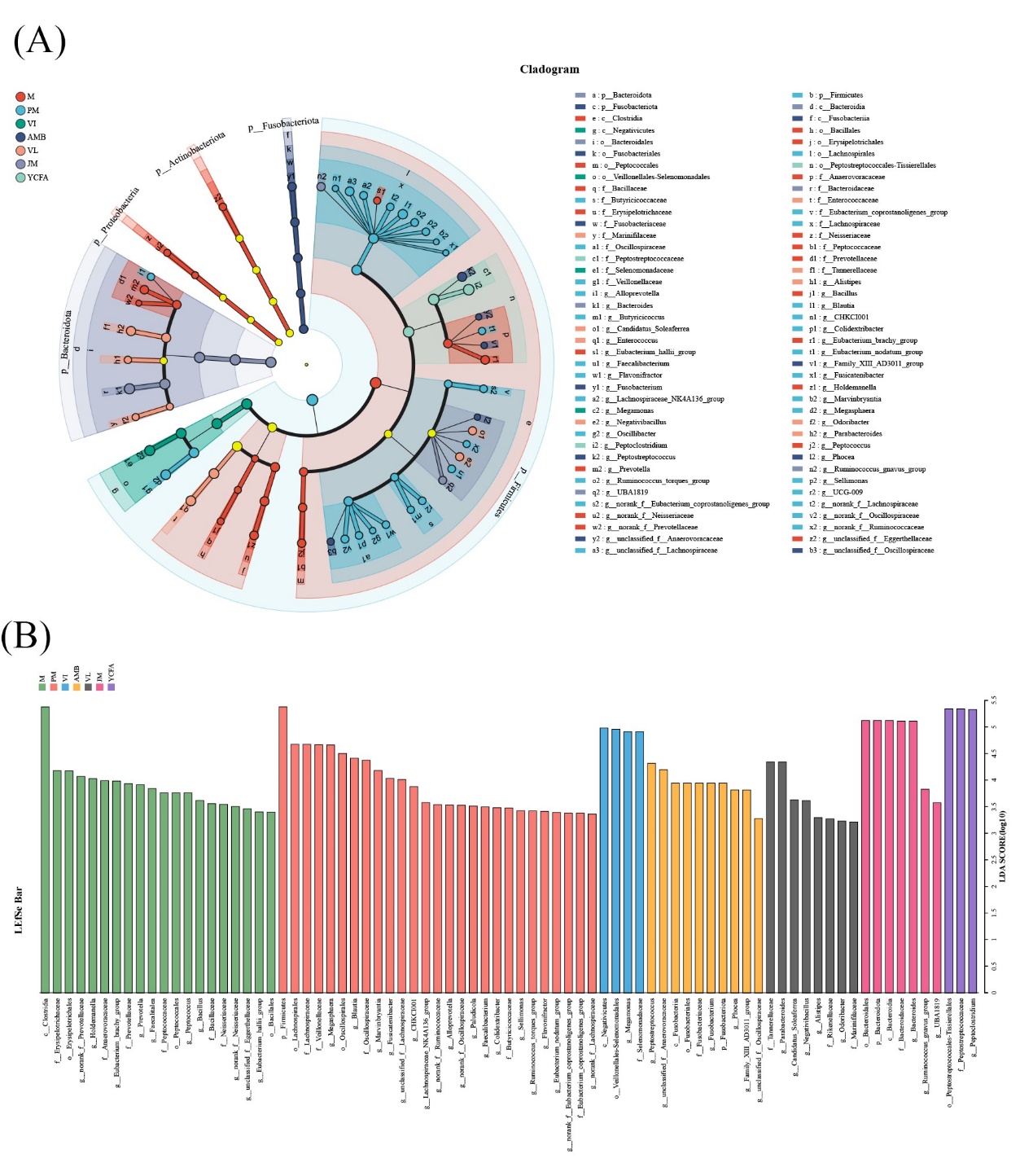


Fig S2 (A) Taxonomic representation of the 8 groups demonstrating statistically and biologically significant differences; (B) Histogram of LDA scores (log10) for differentially abundant features among the 8 groups.

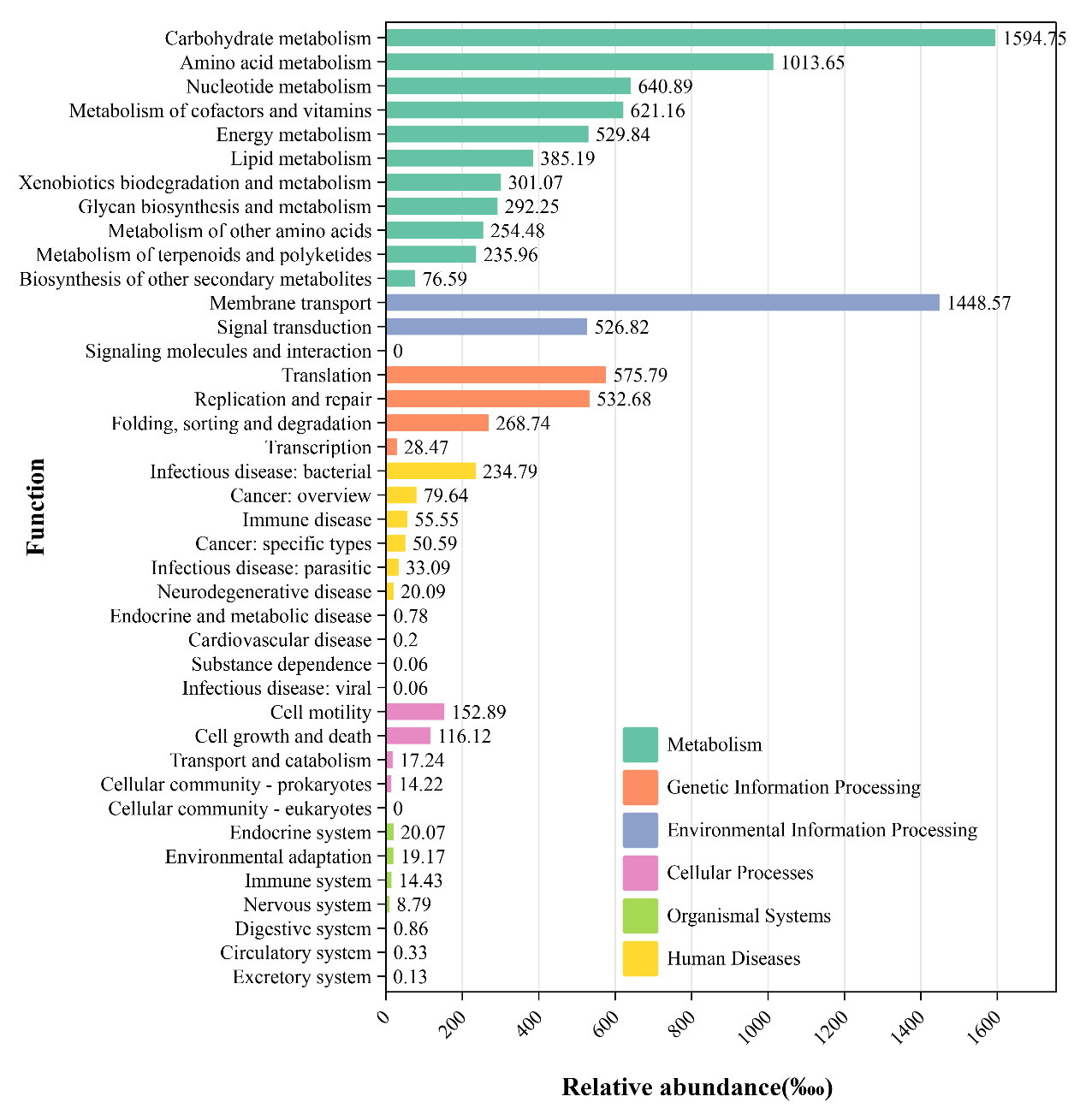


Fig S3. Tax4Fun function prediction of AMB-48h Group (The composition of the pathways of different components is displayed dynamically, the KEGG pathways of different levels are arranged on the vertical axis, and the length of the column indicates the corresponding functional abundance in the pathway.)


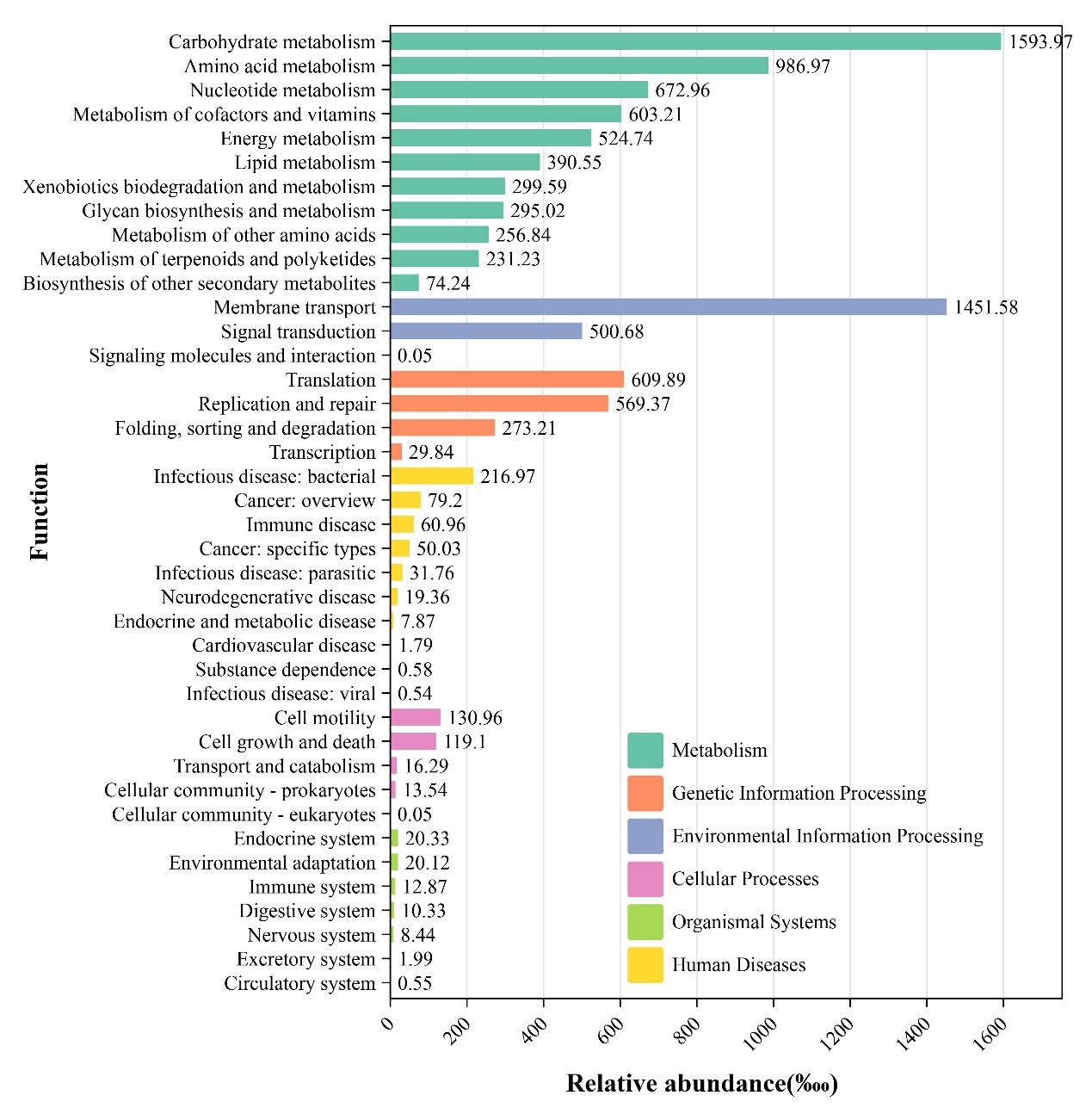


Fig S4. Tax4Fun function prediction of JM-48h Group (The composition of the pathways of different components is displayed dynamically, the KEGG pathways of different levels are arranged on the vertical axis, and the length of the column indicates the corresponding functional abundance in the pathway.)


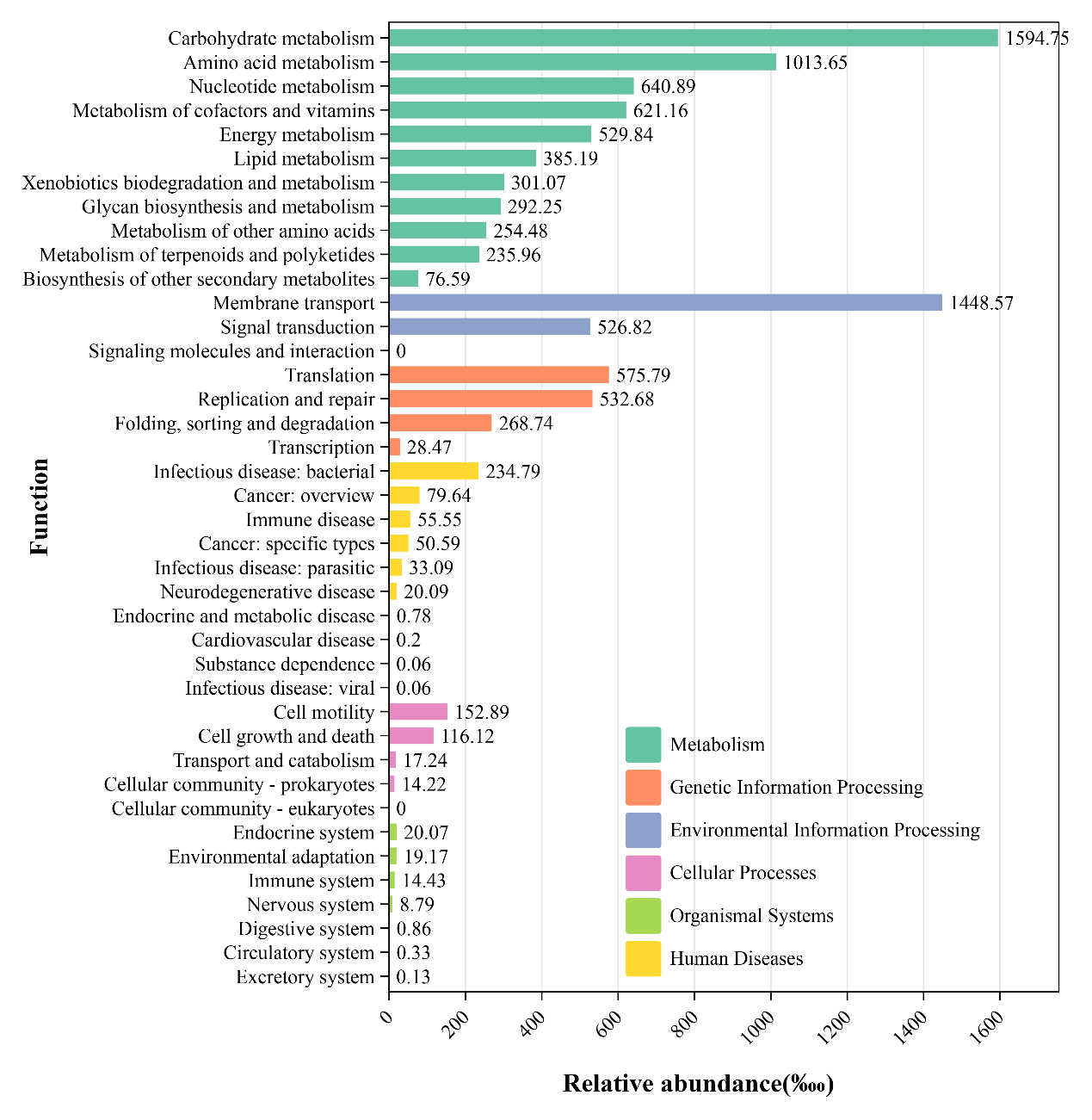


Fig S5. Tax4Fun function prediction of MM-48h Group (The composition of the pathways of different components is displayed dynamically, the KEGG pathways of different levels are arranged on the vertical axis, and the length of the column indicates the corresponding functional abundance in the pathway.)


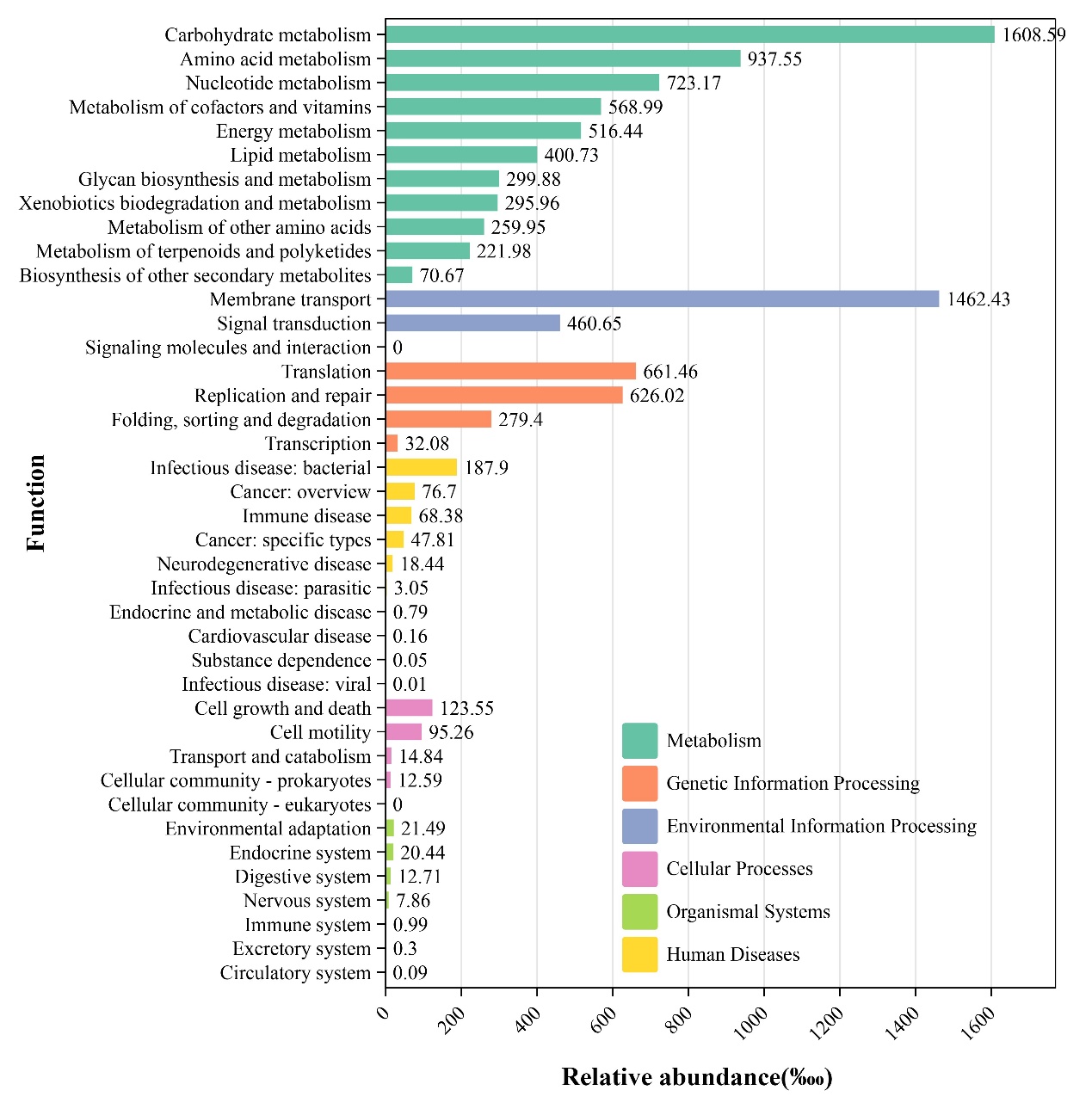


Fig S6. Tax4Fun function prediction of VI-48h Group (The composition of the pathways of different components is displayed dynamically, the KEGG pathways of different levels are arranged on the vertical axis, and the length of the column indicates the corresponding functional abundance in the pathway.)


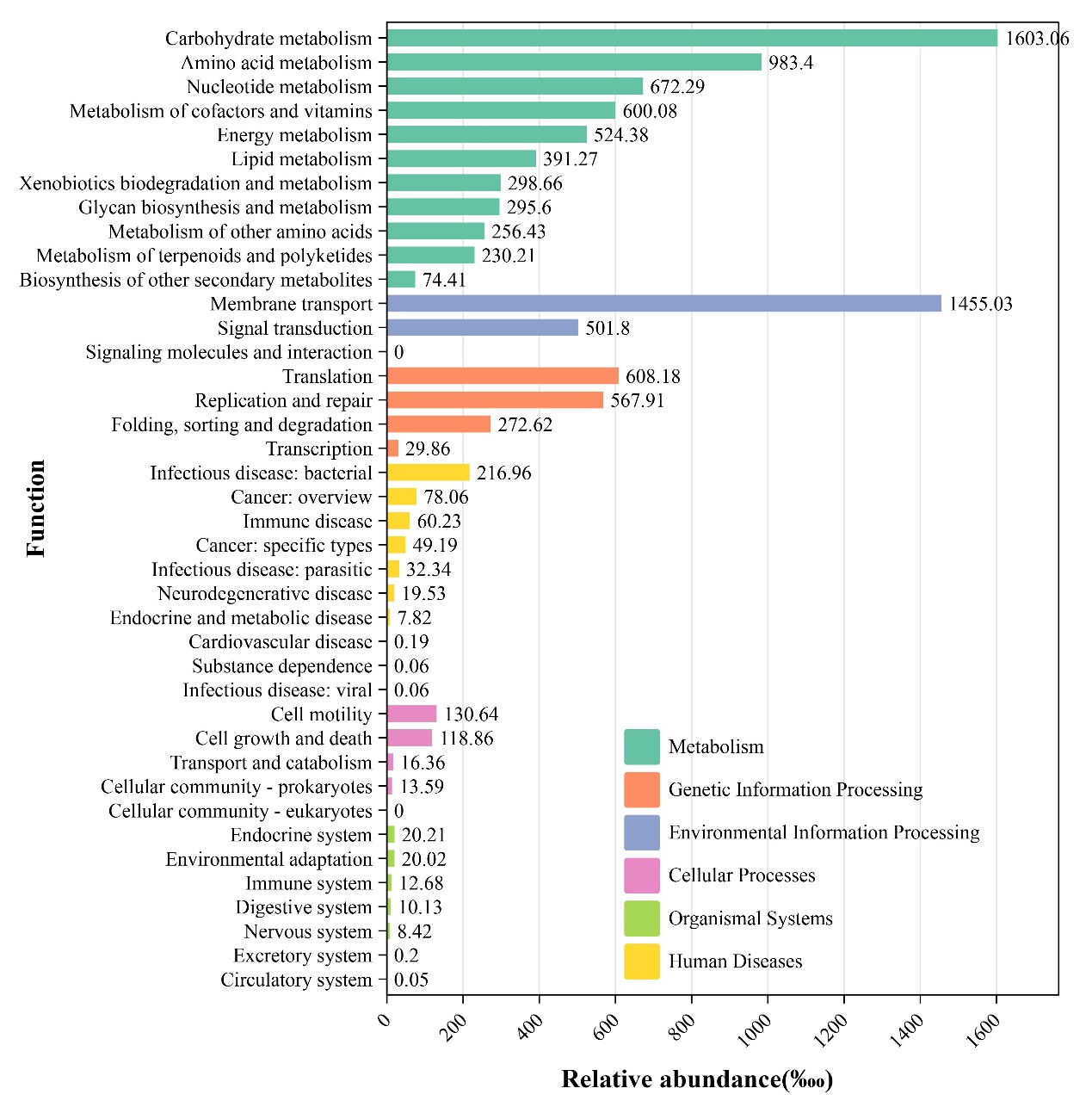


Fig S7. Tax4Fun function prediction of VL-48h Group (The composition of the pathways of different components is displayed dynamically, the KEGG pathways of different levels are arranged on the vertical axis, and the length of the column indicates the corresponding functional abundance in the pathway.)


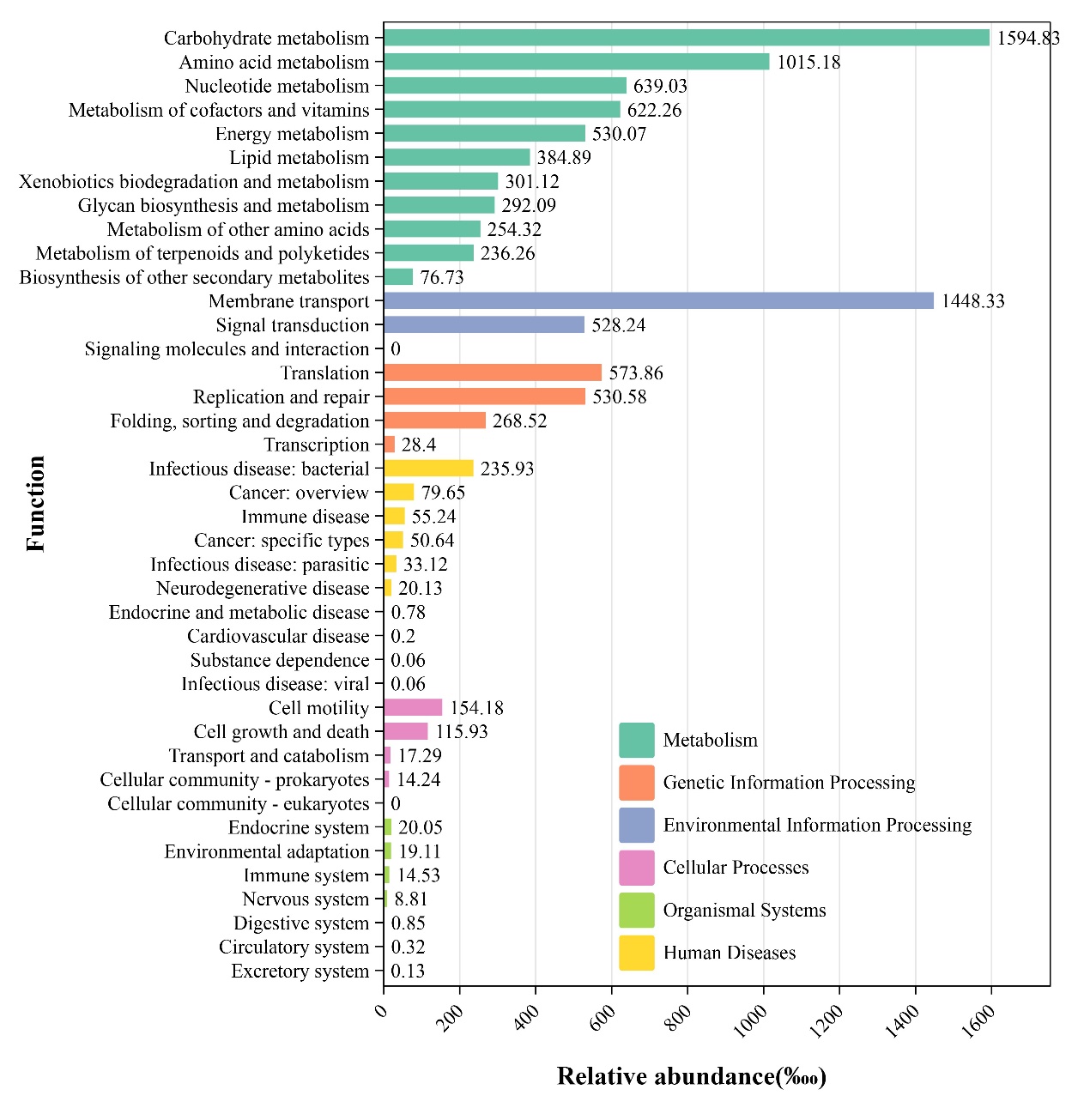


Fig S8. Tax4Fun function prediction of YCFA-48h Group (The composition of the pathways of different components is displayed dynamically, the KEGG pathways of different levels are arranged on the vertical axis, and the length of the column indicates the corresponding functional abundance in the pathway.)


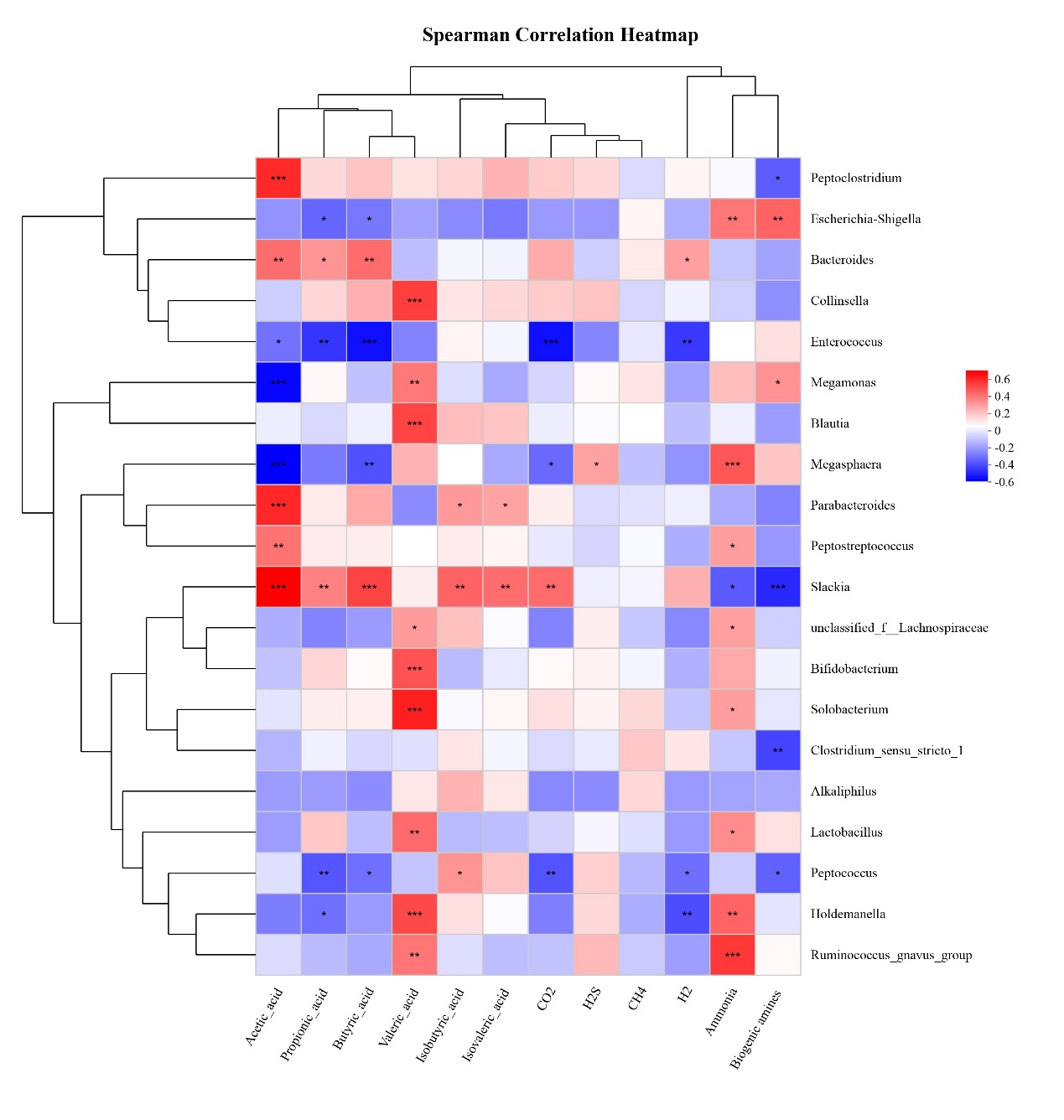


Fig S9. Correlations between metabolites and the GM. The correlation heatmap was measured using the Spearman correlation coefficient. The significance of the correlation is represented by **p* value < 0.05, ***p* value < 0.01, and ****p* value < 0.001.
